# Preformulation Studies of Bee Venom for the Preparation of Bee Venom-Loaded PLGA Particles

**DOI:** 10.3390/molecules200815072

**Published:** 2015-08-18

**Authors:** Min-Ho Park, Ju-Heon Kim, Jong-Woon Jeon, Jin-Kyu Park, Bong-Joo Lee, Guk-Hyun Suh, Cheong-Weon Cho

**Affiliations:** 1 Institute of Drug Research and Development, College of Pharmacy, Chungnam National University, Daejeon 305-764, Korea; E-Mails: parkminho9@daum.net (M.-H.P.); moom5611@naver.com (J.-H.K.); 2 Wissen Co., Ltd, #410 Bio Venture Town, 461-8, Daejeon 305-811, Korea; E-Mails: confessor@hanmail.net (J.-W.J.); jkypark@live.co.kr (J.-K.P.); 3 Department of Veterinary Infectious Diseases, College of Veterinary Medicine, Chonnam National University, Gwangju 500-757, Korea; E-Mail: bjlee@chonnam.ac.kr; 4 Department of Veterinary Internal Medicine, College of Veterinary Medicine, Chonnam National Univeristy, Gwangju 500-757, Korea; E-Mail: ghsuh@chonnam.ac.kr

**Keywords:** bee venom, preformulation, PLGA nanoparticle, sustained release

## Abstract

It is known that allergic people was potentially vulnerable to bee venom (BV), which can induce an anaphylactic shock, eventually leading to death. Up until recently, this kind of allergy was treated only by venom immunotherapy (VIT) and its efficacy has been recognized worldwide. This treatment is practiced by subcutaneous injections that gradually increase the doses of the allergen. This is inconvenient for patients due to frequent injections. Poly (D,L-lactide-co-glycolide) (PLGA) has been broadly studied as a carrier for drug delivery systems (DDS) of proteins and peptides. PLGA particles usually induce a sustained release. In this study, the physicochemical properties of BV were examined prior to the preparation of BV-loaded PLGA nanoparticles NPs). The content of melittin, the main component of BV, was 53.3%. When protected from the light BV was stable at 4 °C in distilled water, during 8 weeks. BV-loaded PLGA particles were prepared using dichloromethane as the most suitable organic solvent and two min of ultrasonic emulsification time. This study has characterized the physicochemical properties of BV for the preparation BV-loaded PLGA NPs in order to design and optimize a suitable sustained release system in the future.

## 1. Introduction

**Figure 1:**
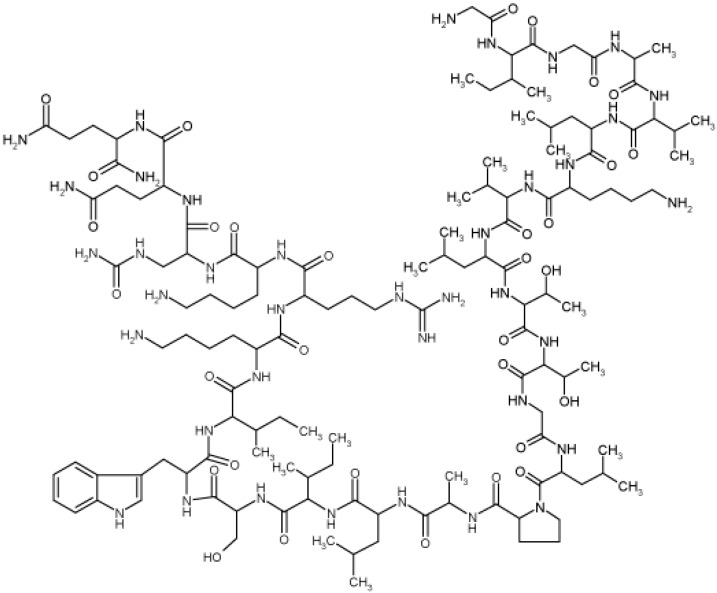
Chemical structure and amino acid sequence of bee venom.

BV comes from the sting of honey bees that use it to protect the bee colony. BV is a protein complex consisting of melittin, phospholipase A2, apamin, and hyaluronidase. Among these components, melittin (Figure 1), a 26 amino acid peptide with the following sequence: Gly-Ile-Gly-Ala-Val-Leu-Lys-Val-Leu-Thr-Thr-Gly-Leu-Pro-Ala-Leu-Ile-Ser-Trp-Ile-Lys-Arg-Lys-Arg-Gln-Gin-NH_2_ is the principal component of BV [1]. The molecular weight of melittin is about 3 kD. It has a high aqueous solubility, which has potential for use as a biologically active peptide drug. BV had been found to have good effects on the immune system [2], cardiovascular system [3], blood [4], and anti-tumor effects [5]. BV therapy has also been practiced in traditional medicine [6,7,8]. Among the BV components, melittin is commonly used in the treatment of arthritic disorders, such as rheumatoid arthritis and osteoarthritis [9,10,11]. Direct stings using bees was a traditional treatment method for arthritis. It has many disadvantages such as the pain caused by the sting, difficulty for maintaining the regular blood concentration, the need for long-term administration of a series of stings or injections due to the short half-life of melittin, and the inconvenience to patients. The conventional regimen also requires a course of administration three times a week over 8–10 weeks, with a high cost [12]. These disadvantages often lead to poor patient compliance [13], making necessary the design of a suitable sustained release system which provides a long-term and constant therapeutic effect.

**Figure 2:**
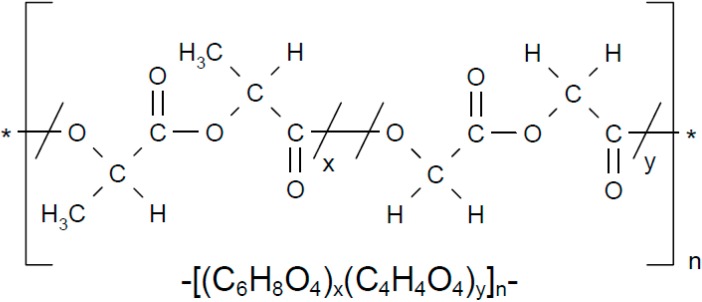
Chemical structured of PLGA types. Chemical structure of PLGA 50:50 (R 502H); Chemical structure of PLGA 75:25 (R 752H).

In recent years, nanoparticles (NPs) were prepared from biodegradable polymers such as poly-d,l-lactic-co-glycolic acid (PLGA). PLGA (Figure 2), a copolymer of lactic and glycolic acid approved by FDA for certain clinical uses, is one of the most biocompatible, biodegradable and non-toxic materials used for preparing NPs [14]. It has the ability to control the release of active pharmaceutical ingredient from NPs, resulting in improved patient compliance by eliminating the need for frequent injections [15,16]. The degradation period of NP can be controlled from days to years by altering the type and amount of polymer, the polymer molecular weight, or the NP structure. PLGA particles have also been demonstrated to be an excellent drug carrier for arthritic lesions following radiopharmaceutical scintigraphic studies in rabbits [17]. BV can be sustain-released and maintained its efficacy for a long time using PLGA particles, which decreases the administration interval and improves patient compliance. It is essential to characterize the physicochemical properties of BV prior to preparation of BV-loaded PLGA particles. Therefore, the aim of this study was to characterize the physicochemical properties of BV under the various conditions related with the preparation of PLGA particles.

## 2. Results and Discussion

### 2.1. Determination of Melittin Content in the BV

Melittin was selected as a marker compound in BV, because it is known that melittin is the most abundant component among the complex of proteins and peptides that constitute BV. In this study we found that the content of melittin was 53.3% ± 0.9%. This result is similar to those found in other publications [7,18].

### 2.2. Stability of BV

Three common protein degradation pathways are aggregation, deamidation, and oxidation. The factors affecting degradation are various, such as pH, ionic strength, temperature, buffer composition and so on. Strategies to reduce protein degradation are based on an understanding of the degradation mechanisms, the changes in the storage conditions, and the effect of formulation component changes on the degradation [19,20], so we conducted stability tests on BV according to storage conditions, pH, ultrasonication, homogenization, and exposure to organic solvents to investigate the stability of BV for the preparation of BV-loaded PLGA particles in the future.

**Figure 3:**
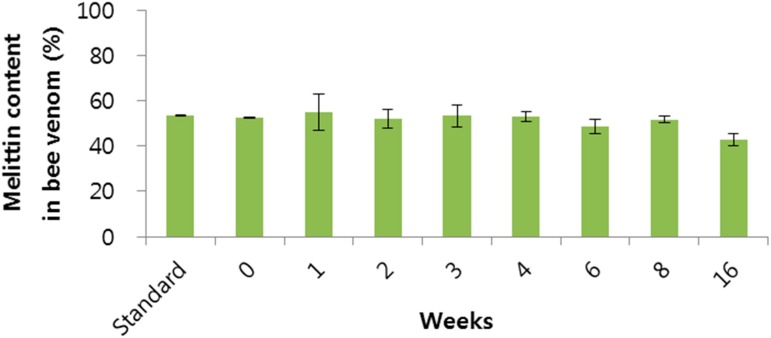
The melittin content in bee venom according to storage period. Data are expressed as the mean ± SD (*n* = 3).

The stability of BV might be affected by the storage conditions and storage period. We thought the most stable storage condition for BV could be at 4 °C in distilled water, with shading from light except for dry powder. The amount of melittin according to storage period is shown in Figure 3. A significant decrease of 10% melittin was observed over 8 weeks. Based on this result, it was suggested that the stability of melittin at 4 °C in distilled water, with protection from light, could be maintained for up to 8 weeks.

**Figure 4:**
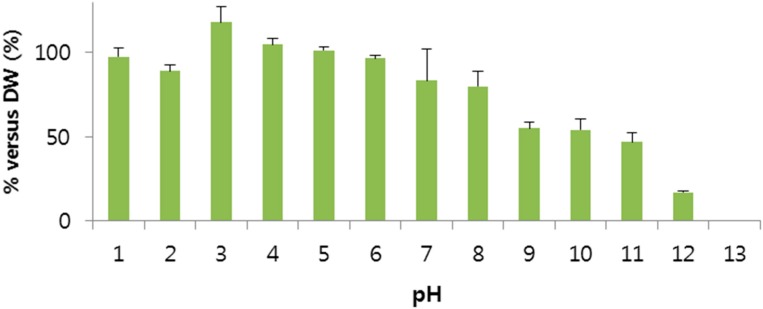
Effect of pH on bee venom. The bee venom was stable in acid condition (pH 1–6). Data are expressed as the mean ± SD (*n* = 3).

Generally, pH has a strong effect on protein aggregation. Proteins are often stable against aggregation over narrow pH ranges and may aggregate rapidly in solutions with pH outside these ranges [21,22,23,24]. To investigate the effect of pH on the BV, this experiment was performed using the various pH solutions. The effect of pH on the BV is shown in Figure 4. The BV was stable under acid condition (pH 1–6), but was less stable under neutral conditions and unstable under basic conditions. From this result, we knew the BV is stable and the PLGA particle preparation process usually occurs at acidic conditions under pH 6. Many papers [25,26,27] explain how the electrostatic charge repulsion destabilizes the tetrameric helical structure of melittin. A low pH decreases the electrostatic charge repulsion and thus stabilizes the conformational stability of melittin.

Organic solvents used for the preparation of NPs may denature proteins. Metabolites and acids liberated by the degrading poly(ester) degrade proteins, which causes insoluble protein agglomeration and leads to incomplete protein release [28,29,30,31].

**Figure 5:**
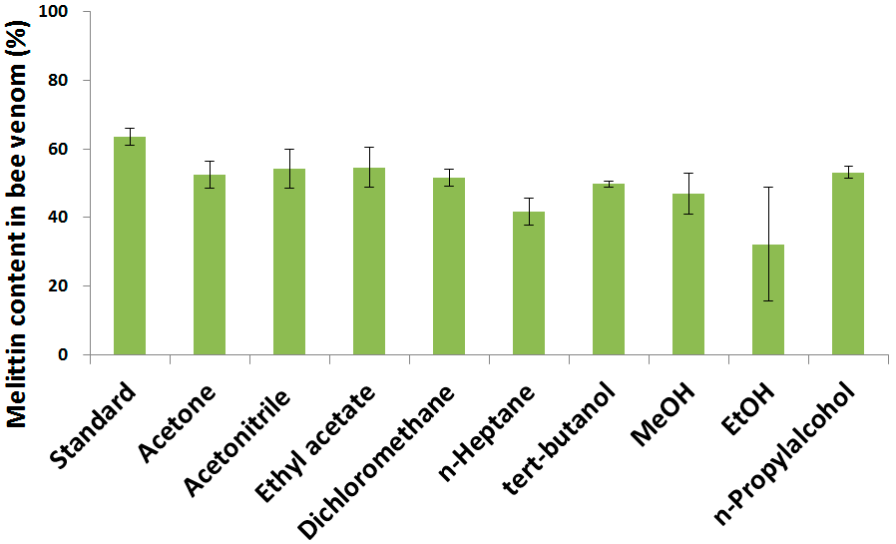
Effect of organic solvents on bee venom; Data are expressed as the mean ± SD (*n* = 3).

To select an adequate organic solvent and investigate the effect of organic solvent on BV during the preparation of BV-loaded PLGA particles, this experiment was performed using various organic solvents. The effect of the various organic solvents on the BV is shown in Figure 5. The melittin content in BV decreased by 10% in all organic solvents. It was thought that the effect of the organic solvents was minimal due to the short contact time between the BV and organic solvent during the actual preparation process, which could be prevented using sugars [32]. The melittin showed below 50% stability in *n*-heptane and ethanol and above 50% in acetonitrile, ethyl acetate, dichloromethane, and *n*-propyl alcohol. Among these solvents, we think dichloromethane is the most suitable organic solvent for the preparation of BV-loaded PLGA particles because acetonitrile is too expensive to be used as organic solvent for PLGA particle preparation and the yield is very low. In addition, agglutination of particles was occurred in ethyl acetate and *n*-propyl alcohol (data not shown).

**Figure 6:**
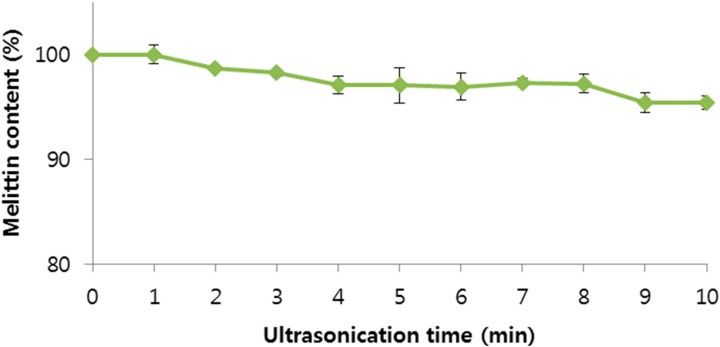
Effect of ultrasonication time on bee venom. Data are expressed as the mean ± SD (*n* = 3).

Ultrasonic and homogenization emulsification methods have been studied for many decades and have attracted increasing interest recently [33,34]. Studies comparing ultrasonic emulsification with rotor-stator dispersing [34,35] found ultrasound to be competitive or even superior in terms of droplet size and energetic efficiency. Ultrasonication and homogenization efficiently homogenize aqueous protein solutions in organic polymer solutions, which is the first important step for efficient drug encapsulation in PLGA particles. However, ultrasonication, like other homogenization methods, can damage the physical stability of peptides and proteins, thus the effect of ultrasonication on BV stability was examined by ultrasonicating BV solutions. Ultrasonication times over 2 min led to a statistically significant decrease of melittin concentration (*p* < 0.05) compared to 0 min, as determined by *t*-test (Figure 6). Ultrasonication times over 2 min might damage the physical stability of BV. Based on the result, the time under 2 min was selected as ultrasonic emulsification time for the preparation of BV-loaded PLGA particles, in agreement with the literature [36].

**Figure 7:**
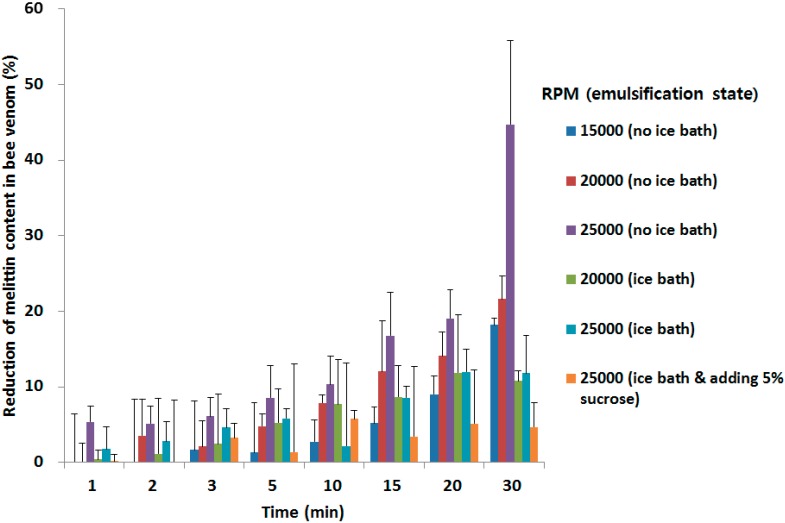
Effect of homogenization time and state on bee venom. Data are expressed as the mean ± SD (*n* = 3).

The effect of homogenization on BV stability was also examined by homogenizing BV solutions (Figure 7).

The higher the RPM of the homogenizer, the greater the reduction of melittin content in BV was. We thought the strong physical power and heat generated by the homogenizer damaged the BV. In the view of heat produced by the homogenizer, the stability of BV might be improved by using an ice bath during the homogenization. Also, sucrose improved the stability of BV during the homogenization and this was correlated with the fact that hydration by sucrose increases protein. Sucrose surrounds BV and it protect from the physical power generated by the homogenizer. It is agreed with other publication, protecting the protein from the physical power generated by the homogenizer [37].

### 2.3. Surface Morphology of BV

**Figure 8:**
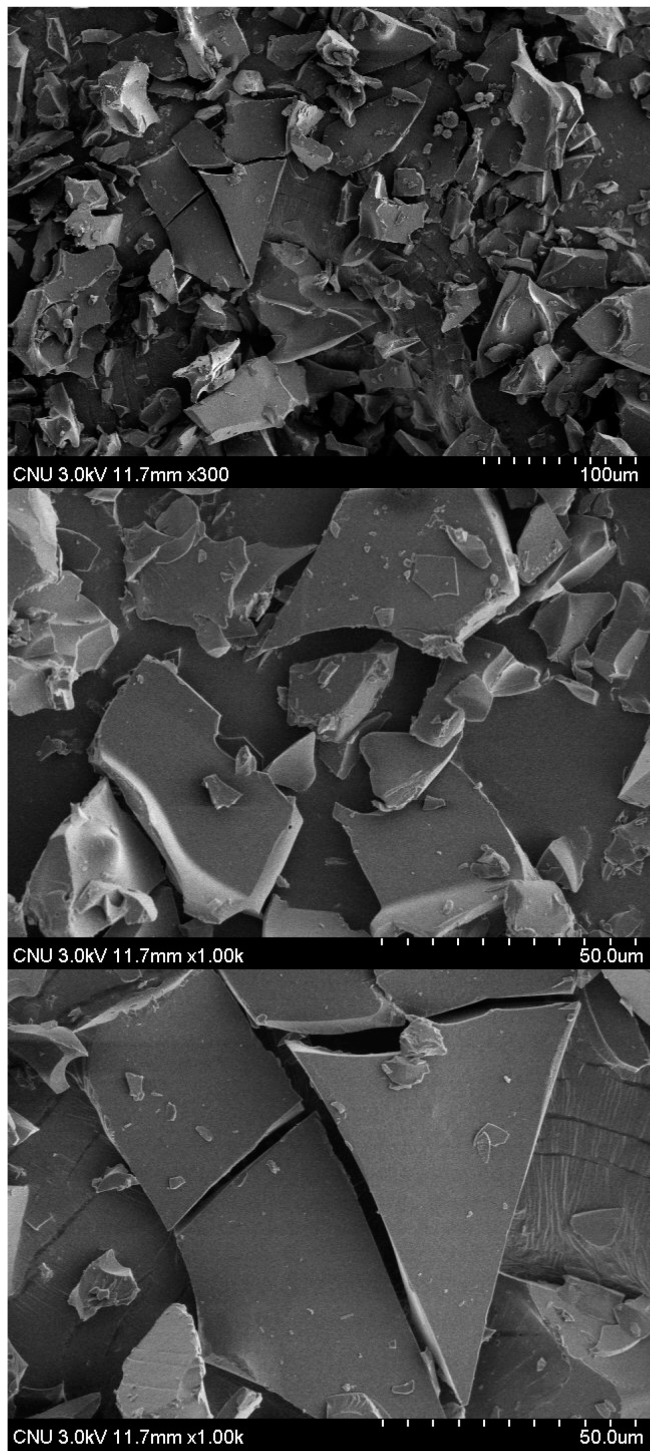
Surface morphology of bee venom.

SEM was used to assess the microscopic surface morphology of the drug. BV is characterized by the presence of irregular sized particles. Photomicrographs of the samples obtained by SEM are shown in Figure 8. The surface of BV was smooth and irregular like a piece of broken glass. This might be because BV is a complex of proteins. The SEM images showed BV was in a crystal form and did not have protein aggregation, meaning BV was stable in the solid state.

## 3. Experimental Section 

### 3.1. Materials

BV was obtained from Wissen Co. Ltd (Daejeon, Korea). PLGA (50:50 Resomer RG 502H and 75:25 Resomer RG 752H) with free carboxyl end groups was a gift from Dongkook Pharm (Jincheon, Korea). Polyvinyl alcohol (PVA) (M.W; 13,000–23,000 and 146,000–186,000) and a melittin standard were purchased from Sigma-Aldrich Korea (Yongin, Korea). Ethyl acetate was purchased from Daejung Chemicals and Materials (Daejeon, Korea). Dichloromethane (DCM) was purchased from Samchun Chemicals (Pyeongtaek, Korea). All other chemicals were commercial analytical grade products and used without further purification.

### 3.2. High Performance Liquid Chromatography (HPLC)

Melittin was selected as an marker compound in BV because it is the most abundant component among the complex of proteins in BV. Stock solution of melittin was prepared at 2 mg/mL in distilled water. To prepare calibration standards, this stock solution was serially diluted using distilled water and yielded 1000, 500, 250, 125, 50, 25, 5, 1 µg/mL solutions for linearity and lower limit of quantification (LLOQ) assays. The concentration of melittin was determined using an Agilent 1100 Series HPLC (Agilent Technologies, Palo Alto, CA, USA) instrument, operated at ambient temperature, consisting of an automatic autosampler system equipped with a loop injection valve and a variable wavelength UV-VIS detector. The column used was a C18 column (Zobrax, 250 × 4.6 mm, 5 µm particle size, Agilent^®^). Column temperature was 35 °C. The flow rate of the mobile phase was 1 mL/min and the detection wavelength was set to 280 nm. The mobile phase A was 0.1% trifluoroacetic acid in distilled water containing 10% acetonitrile. The mobile phase B was 0.1% trifluoroacetic acid in acetonitrile containing 10% distilled water. The mobile phase was filtered through a 0.45 μm membrane filter and degassed via ultrasonic water bath prior to use. The following gradient conditions were used: 75% mobile phase A for 3 min, from 75% to 50% mobile phase A in 3 min, from 50% to 20%, back to 75% mobile phase A in 4 min, and maintain mobile phase A for 5 min. Injection volume was 20 μL.

### 3.3. Characterizations of BV

#### 3.3.1. Determination of Melittin Content in the BV

BV was dissolved in distilled water at a concentration of 1 mg/mL. The BV solution was centrifuged at 4 °C and 12,000 rpm for 10 min for HPLC. The HPLC peak area of melittin in the BV was compared with that of a melittin standard.

#### 3.3.2. Stability Test of BV

A stability test of melittin in BV on storage at 4 °C in distilled water, shaded from light, was conducted. Samples were taken at indicated intervals of 0, 1, 2, 3, 4, 6, 8 and 16 weeks from three batches. As well as, the stability test of melittin in BV according to pH was conducted in various pH values. BV was added to the various pH solutions (1 mg/mL) and stirred for 72 h. The amount of melittin in the samples was then measured by HPLC.

The stability test of melittin in BV in organic solvent was conducted by adding BV to various organic solvents (glacial acetic acid, acetone, acetonitrile, ethyl acetate, dichloromethane, *n*-heptane, *tert*-butanol, tetrahydrofuran, MeOH, EtOH, *n*-propyl alcohol, 1 mg/mL) and incubating the solutions for 1 h. Samples were stirred sufficiently and the organic solvent was evaporated under a N_2_ gas purge. Distilled water was added to the samples and the samples (1 mg/mL) were stirred. The amount of melittin in the samples was measured by HPLC. A stability test of melittin in BV under ultrasonication was conducted. BV was dissolved in distilled water (0.8 mg/mL). Samples were ultrasonicated at 8–12 W for 10 min. The amount of melittin of each sample was then measured by HPLC. In addition, the stability melittin in BV towards RPM and time of homogenization was tested. BV was dissolved in distilled water (1 mg/mL). Samples were homogenized at 15,000, 20,000, and 25,000 rpm under no ice bath, ice bath, or ice bath adding 5% sucrose conditions. Samples (0.8 mL) were obtained at 1, 2, 3, 5, 10, 15, 20, and 30 min and the amount of melittin in the sample was measured by HPLC.

#### 3.3.3. Observation of BV Surface Morphology

Scanning electron microscopy (SEM) was used to observe the shape of BV. BV was dropped onto double-sided carbon tape, which was then vacuum-coated for 50 seconds with a mixture of gold and palladium and the morphology examined with a FE-SEM (JEOL JSM7500, Thermo, Waltham, MA, USA) at 3 kV accelerating voltage.

## 4. Conclusions

In this study, we characterized the physicochemical properties of BV under various conditions related to the preparation of PLGA particles. We examined the various effects of organic solvent, content of melittin in the BV, pH, ultrasonication time, and emulsification time and rate and stability of BV in PLGA particles. The concentration of melittin, the main component of BV was 53.3%. The stability of BV at 4 °C in distilled water was maintained during 8 weeks when shaded from the light. Among organic solvents, dichloromethane was the most suitable for the preparation of BV-loaded PLGA particle. Two min was selected as the ultrasonic emulsification time for the preparation of BV-loaded PLGA particles. Based on the results, this study provides experimental parameters for the preparation of BV-loaded PLGA particles. Further studies will be performed such as the actual preparation of BV-loaded PLGA particles, optimization of the preparation process, *in vitro* release tests, and *in vivo* use tests.

## References

[1] Niemz A., Tirrell D.A. (2001). Self-association and membrane-binding behavior of melittins containing trifluoroleucine. J. Am. Chem. Soc..

[2] Jutel M., Pichler W.J., Skrbic D., Urwyler A., Dahinden C., Muller U.R. (1995). Bee Venom Immunotherapy Results in Decrease of IL-4 and IL-5 and Increase of Ifn-Gamma Secretion in Specific Allergen-Stimulated T-Cell Cultures. J. Immunol..

[3] Thomas G.R., Hiley C.R. (1988). Cardiovascular Effects of Intracerebro-Ventricular Bradykinin and Melittin in the Rat. J. Pharm. Pharmacol..

[4] Pogliani E.M., Cofrancesco E. (1983). Thrombotic thrombocytopenic purpura: A review. Haematologica.

[5] Liu X., Chen D., Xie L., Zhang R. (2002). Effect of honey bee venom on proliferation of K1735M2 mouse melanoma cells *in vitro* and growth of murine B16 melanomas *in vivo*. J. Pharm. Pharmacol..

[6] Billingham M.E., Morley J., Hanson J.M., Shipolini R.A., Vernon C.A. (1973). Letter: An anti-inflammatory peptide from bee venom. Nature.

[7] Hider R.C. (1988). Honeybee venom: A rich source of pharmacologically active peptides. Endeavour.

[8] Liu H., Tong F. (2003). Advances in the study of bee venom and its clinical uses. Zhong Yao Cai.

[9] Eiseman J.L., von Bredow J., Alvares A.P. (1982). Effect of honeybee (*Apis mellifera*) venom on the course of adjuvant-induced arthritis and depression of drug metabolism in the rat. Biochem. Pharmacol..

[10] Hadjipetroukourounakis L., Yiangou M. (1988). Bee Venom, Adjuvant Induced Disease and Interleukin Production. J. Rheumatol..

[11] Lee J.Y., Kang S.S., Kim J.H., Bae C.S., Choi S.H. (2005). Inhibitory effect of whole bee venom in adjuvant-induced arthritis. In Vivo.

[12] Durham S.R., Walker S.M., Varga E.M., Jacobson M.R., O’Brien F., Noble W., Till S.J., Hamid Q.A., Nouri-Aria K.T. (1999). Long-term clinical efficacy of grass-pollen immunotherapy. N. Engl. J. Med..

[13] Qiao M., Chen D., Hao T., Zhao X., Hu H., Ma X. (2007). Effect of bee venom peptide-copolymer interactions on thermosensitive hydrogel delivery systems. Int. J. Pharm..

[14] Bala I., Hariharan S., Kumar M.N. (2004). PLGA nanoparticles in drug delivery: The state of the art. Crit. Rev. Ther. Drug Carrier Syst..

[15] Jones A.J., Putney S., Johnson O.L., Cleland J.L. (1997). Recombinant human growth hormone poly(lactic-co-glycolic acid) microsphere formulation development. Adv. Drug Deliv. Rev..

[16] Ogawa Y., Yamamoto M., Okada H., Yashiki T., Shimamoto T. (1988). A New Technique to Efficiently Entrap Leuprolide Acetate into Microcapsules of Polylactic Acid or Copoly(Lactic Glycolic) Acid. Chem. Pharm. Bull..

[17] Tuncay M., Calis S., Kas H.S., Ercan M.T., Peksoy I., Hincal A.A. (2000). Diclofenac sodium incorporated PLGA (50:50) microspheres: Formulation considerations and *in vitro*/*in vivo* evaluation. Int. J. Pharm..

[18] Peiren N., Vanrobaeys F., de Graaf D.C., Devreese B., van Beeumen J., Jacobs F.J. (2005). The protein composition of honeybee venom reconsidered by a proteomic approach. Biochim. Biophys. Acta.

[19] Cleland J.L., Powell M.F., Shire S.J. (1994). The Development of Stable Protein Formulations: A Close Look at Protein Aggregation, Deamidation, and Oxidation. Crit. Rev. Ther. Drug Carrier Syst..

[20] Van de Weert M., Hennink W.E., Jiskoot W. (2000). Protein instability in poly(lactic-co-glycolic acid) microparticles. Pharm. Res..

[21] Fatouros A., Osterberg T., Mikaelsson M. (1997). Recombinant factor VIII SQ—Influence of oxygen, metal ions, pH and ionic strength on its stability in aqueous solution. Int. J. Pharm..

[22] Kerwin B.A., Akers M.J., Apostol I., Moore-Einsel C., Etter J.E., Hess E., Lippincott J., Levine J., Mathews A.J., Revilla-Sharp P. (1999). Acute and long-term stability studies of deoxy hemoglobin and characterization of ascorbate-induced modifications. J. Pharm. Sci..

[23] Nielsen L., Khurana R., Coats A., Frokjaer S., Brange J., Vyas S., Uversky V.N., Fink A.L. (2001). Effect of environmental factors on the kinetics of insulin fibril formation: Elucidation of the molecular mechanism. Biochemistry.

[24] Townsend M.W., DeLuca P.P. (1990). Stability of ribonuclease A in solution and the freeze-dried state. J. Pharm. Sci..

[25] Bello J., Bello H.R., Granados E. (1982). Conformation and aggregation of melittin: Dependence on pH and concentration. Biochemistry.

[26] Goto Y., Hagihara Y. (1992). Mechanism of the conformational transition of melittin. Biochemistry.

[27] Hagihara Y., Kataoka M., Aimoto S., Goto Y. (1992). Charge repulsion in the conformational stability of melittin. Biochemistry.

[28] Aubert-Pouessel A., Venier-Julienne M.C., Saulnier P., Sergent M., Benoit J.P. (2004). Preparation of PLGA microparticles by an emulsion-extraction process using glycofurol as polymer solvent. Pharm. Res..

[29] Chi E.Y., Krishnan S., Randolph T.W., Carpenter J.F. (2003). Physical stability of proteins in aqueous solution: Mechanism and driving forces in nonnative protein aggregation. Pharm. Res..

[30] Sinha V.R., Trehan A. (2003). Biodegradable microspheres for protein delivery. J. Control. Release.

[31] Whitaker M.J., Hao J.Y., Davies O.R., Serhatkulu G., Stolnik-Trenkic S., Howdle S.M., Shakesheff K.M. (2005). The production of protein-loaded microparticles by supercritical fluid enhanced mixing and spraying. J. Control. Release.

[32] Arakawa T., Timasheff S.N. (1982). Stabilization of protein structure by sugars. Biochemistry.

[33] Maa Y.F., Hsu C.C. (1999). Performance of Sonication and microfluidization for liquid-liquid emulsification. Pharm. Dev. Technol..

[34] Medina J., Salvado A., del Pozo A. (2001). Use of ultrasound to prepare lipid emulsions of lorazepam for intravenous injection. Int. J. Pharm..

[35] Abismail B., Canselier J.P., Wilhelm A.M., Delmas H., Gourdon C. (1999). Emulsification by ultrasound: Drop size distribution and stability. Ultrason. Sonochem..

[36] Adami R.C., Collard W.T., Gupta S.A., Kwok K.Y., Bonadio J., Rice K.G. (1998). Stability of peptide condensed plasmid DNA formulations. J. Pharm. Sci..

[37] Park S.J., Choi S.G., Davaa E., Park J.S. (2011). Encapsulation enhancement and stabilization of insulin in cationic liposomes. Int. J. Pharm..

